# Irisin preserves mitochondrial integrity and function in tubular epithelial cells after ischemia–reperfusion-induced acute kidney injury

**DOI:** 10.1111/apha.14211

**Published:** 2024-07-29

**Authors:** Yu Cui, Lu Yu, Wenqi Cong, Shan Jiang, Xingyu Qiu, Chunchun Wei, Gui Zheng, Jianhua Mao, Ruisheng Liu, Andreas Patzak, Pontus B. Persson, Jianghua Chen, Liang Zhao, En Yin Lai

**Affiliations:** 1 Kidney Disease Center of the First Affiliated Hospital, Zhejiang University School of Medicine, Hangzhou, China; 2 Key Laboratory of Kidney Disease Prevention and Control Technology, Zhejiang, Hangzhou, China; 3 Zhejiang Clinical Research Center of Kidney and Urinary System Disease, Hangzhou, China; 4 Department of Physiology, School of Basic Medical Sciences, Zhejiang University School of Medicine, Hangzhou, China; 5 Provincial Key Laboratory of Neonatal Diseases, Department of Nephrology, National Clinical Research Center for Child Health, Children's Hospital, Zhejiang University School of Medicine, Hangzhou, China; 6 Department of Molecular Pharmacology & Physiology, Hypertension and Kidney Research Center, Morsani College of Medicine, University of South Florida, Tampa, USA; 7 Institute of Translational Physiology, Charité–Universitätsmedizin Berlin, Freie Universität Berlin and Humboldt-Universität zu Berlin, Berlin, Germany

**Keywords:** acute kidney injury, inflammasome activation, irisin, mitochondrial autophagy, renal ischemia–reperfusion

## Abstract

**Aims:**

A myokine secreted by skeletal muscles during exercise called irisin mitigates ischemia–reperfusion (I/R) injury in epithelial cells of various organs by limiting damage to mitochondria. We test whether irisin may preserve the mitochondrial integrity and function in renal tubular epithelial cells and protect against ischemia–reperfusion-induced acute kidney injury (AKI).

**Methods:**

We correlated serum irisin levels with serum creatinine and BUN levels from both AKI patients and healthy individuals. In mice with irisin administration, various renal injury markers such as serum creatinine, BUN, kidney injury molecule-1 (Kim-1), and neutrophil gelatinase-associated lipocalin (NGAL), and renal histopathology were assessed after I/R. To identify the potential mechanisms of the protective of irisin's protective effect, we perfused proximal tubules under confocal microscopy and analyzed kidney tissues by qPCR, western blot, and immunohistochemistry.

**Results:**

Serum irisin correlated inversely with serum creatinine and BUN levels were significantly lower in AKI patients than in healthy subjects. Administering irisin to mice after I/R decreased biomarker levels for AKI including serum creatinine, BUN, Kim-1, NAGL and lessened histological changes. In kidney tissues of mice, irisin upregulated the mitochondrial autophagy marker protein microtubule-associated protein 1 light chain 3 (LC3), the mitochondrial autophagy pathway-related proteins PTEN-induced putative kinase 1 (PINK1) and Parkinson's disease 2 parkin (PARK2) and downregulated the reactive substrate protein sequestosome 1 (P62) and mitochondrial membrane proteins translocase of outer mitochondrial membrane 20 (TOM20) and translocase of inner mitochondrial membrane 23 (TIM23).

**Conclusion:**

Irisin protects against renal I/R injury, which may involve the preservation of mitochondrial integrity and function.

## 1 ∣ INTRODUCTION

Acute kidney injury (AKI) is a sudden decline in renal function, with high mortality and treatment costs.^1^ The incidence of community-acquired AKI is reaching 10% among outpatients, 20%–30% among inpatients, and can be as high as 50% for intensive care unit (ICU) patients.^2,3^ Hospitalized patients with AKI show high mortality, reaching 50% for those requiring renal replacement therapy.^2^ Surviving AKI patients remain at greater risk for developing chronic kidney disease (CKD).^4,5^ Prompt preventive measures decrease the occurrence and improve the prognosis of AKI. Yet, current treatments for AKI often disappoint.^6^

Skeletal muscles may play a particular role in protecting the kidney from damage. Mild exercise training improves kidney function and is beneficial in renal injury,^7,8^ as is ischemic preconditioning of a remote limb muscle.^9,10^ How the crosstalk between skeletal muscle and kidney occurs is incompletely understood.

Skeletal muscles secrete a multifunctional membrane protein fragment named irisin during exercise.^11,12^ Fibronectin type III domain containing protein 5 (FNDC5) forms blood borne irisin by hydrolyzation and cleavage.^11,13,14^ Several organs express irisin, including the nervous system, heart, kidneys and bone^14,15^ and irisin seems to protect these tissues from ischemic damage.^10,16-19^ Mitochondrial repair and mitochondrial autophagy may play a role in providing protection.^20^ Irisin mitigates ischemia–reperfusion (I/R) injury in epithelial cells of various organs, including the intestine and lungs by improving the mitochondrial function of epithelial cells.^11^

Renal tubular necrosis with secondary inflammatory responses is the key to ischemic AKI. Renal tubular cells have the most abundant mitochondrial content.^21^ Damaged or malfunctioning mitochondria are cleared by mitochondrial autophagy.^19,22,23^ Mitochondrial autophagy limits and repairs organ damage by ischemia and hypoxia.^24^ Thus, we hypothesize that irisin may preserve the mitochondrial integrity and function in renal tubular epithelial cells and protect against I/R-induced AKI.

To test this hypothesis, we measured serum irisin levels and correlated them with serum creatinine among both AKI patients and healthy individuals. We also examined and compared various serum biochemical parameters including creatinine, urea nitrogen (BUN), kidney injury molecule-1 (Kim-1), and neutrophil gelatinase-associated lipocalin (NGAL) alongside renal histopathology in ischemic AKI mice with and without the administration of exogenous irisin. Furthermore, we investigated the mechanisms underlying irisin's effects through microperfusion experiments of renal proximal tubules under confocal microscopy as well as analyses of kidney tissues using techniques such as qPCR, western blot, and immunohistochemistry.

## 2 ∣ RESULTS

### 2.1 ∣ Decreased serum irisin in AKI patients

**FIGURE 1:**
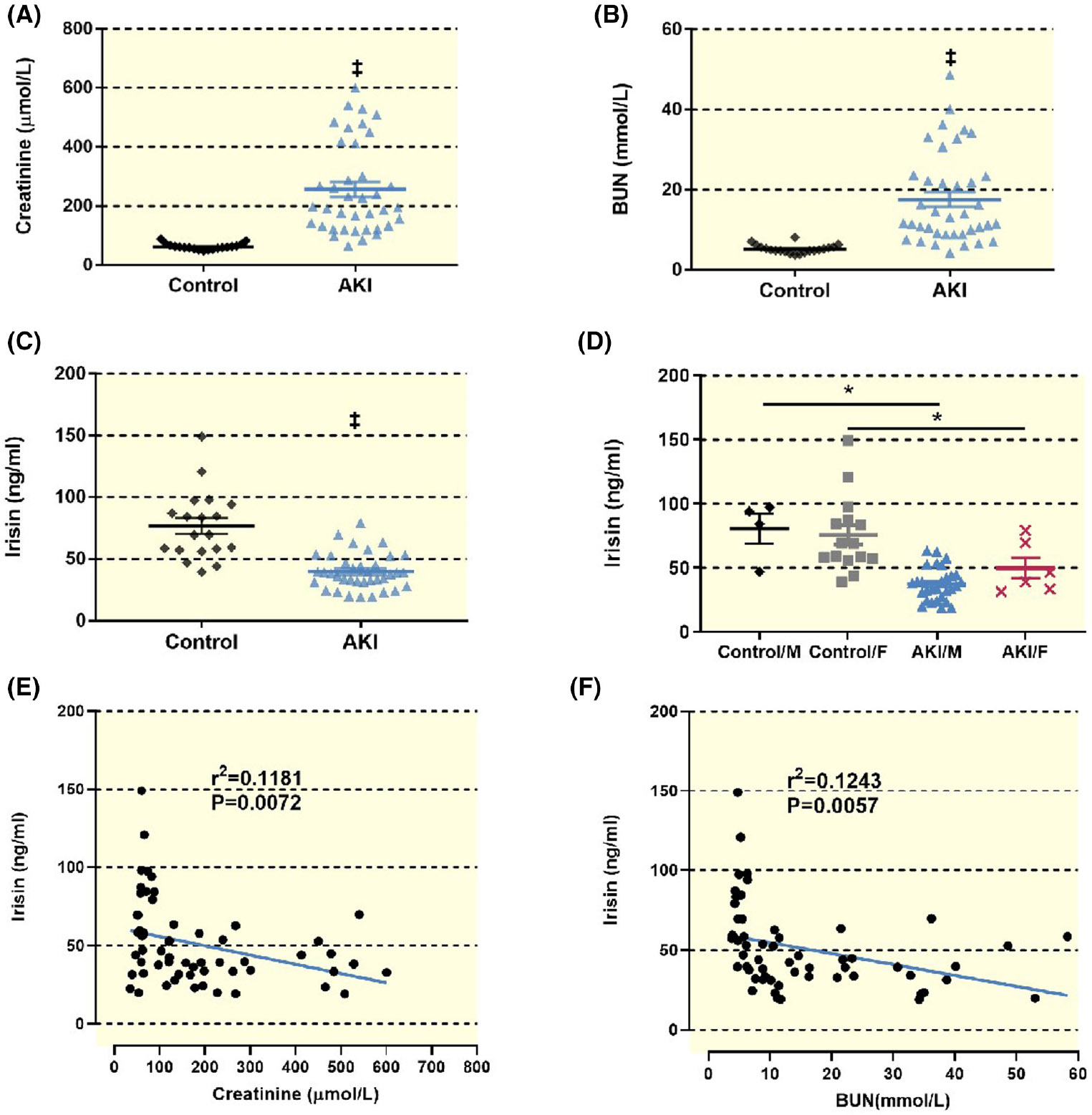
Serum irisin level and their correlation with renal function markers. (A) The serum creatinine level (μmol/L) in the AKI group (*n* = 37) is significantly higher than for the Control group (*n* = 20), (256.8 ± 30.5 vs. 61.9 ± 10.5, ‡*p* < 0.001 vs. Control). (B) The serum BUN level (mmol/L) are greater in the AKI group as compared to the Control group (17.1 ± 6.5 vs. 5.1 ± 1.0, ‡*p* < 0.001 vs. Control). (C) The expression level of serum irisin in the AKI group was significantly lower than that in the Control group (39.9 ± 8.2 vs. 76.7 ± 13.8 ng/mL, ‡*p* < 0.001 vs. Control). Statistical difference in (A–C) was calculated by *t*-test. (D) There is no significant difference between males and females in the Control group and the AKI group. The expression level of serum irisin in male and female of the AKI group was significantly lower than of the Control group respectively (*p < 0.05 vs. Control). (E) There is a linear inverse correlation between serum irisin and serum creatinine levels. The data plotted here are combined data including both AKI group and Control group, *r*^2^ = 0.1181, *p* = 0.0072. (F) There is a linear inverse correlation between serum irisin and serum BUN levels. The data plotted here are combined data including both AKI group and Control group. *r*^2^ = 0.1243, *p* = 0.0057. Statistical difference in (E) and (F) is calculated by Linear Regression.

Levels of serum creatinine and BUN were significantly higher in deceased organ donors who experienced pre-donation AKI patients than those in the Control group (256.8 ± 30.5 vs. 61.9 ± 10.5 μmol/L, *p* < 0.001 and 17.1 ± 6.5 vs. 5.1 ± 1.0 mmol/L, *p* < 0.001). Conversely, serum irisin concentrations were significantly reduced (39.9 ± 8.2 vs. 76.7 ± 13.8 ng/mL, *p* < 0.001; Figure 1A-C). There is no significant difference between males and females in the Control group and the AKI group. The expression level of serum irisin in male and female of the AKI group was significantly lower than of the Control group respectively (Figure 1D). There was a negative linear correlation between serum irisin levels and renal function (*r*^2^ = 0.1181, *p* = 0.0072 in creatinine and *r*^2^ = 0.1243, *p* = 0.0057 in BUN) in patients with ischemic AKI, suggesting that lower serum irisin levels are associated with poorer renal function (Figure 1E,F).

### 2.2 ∣ The therapeutic effect of irisin in ischemic AKI mice model

**FIGURE 2:**
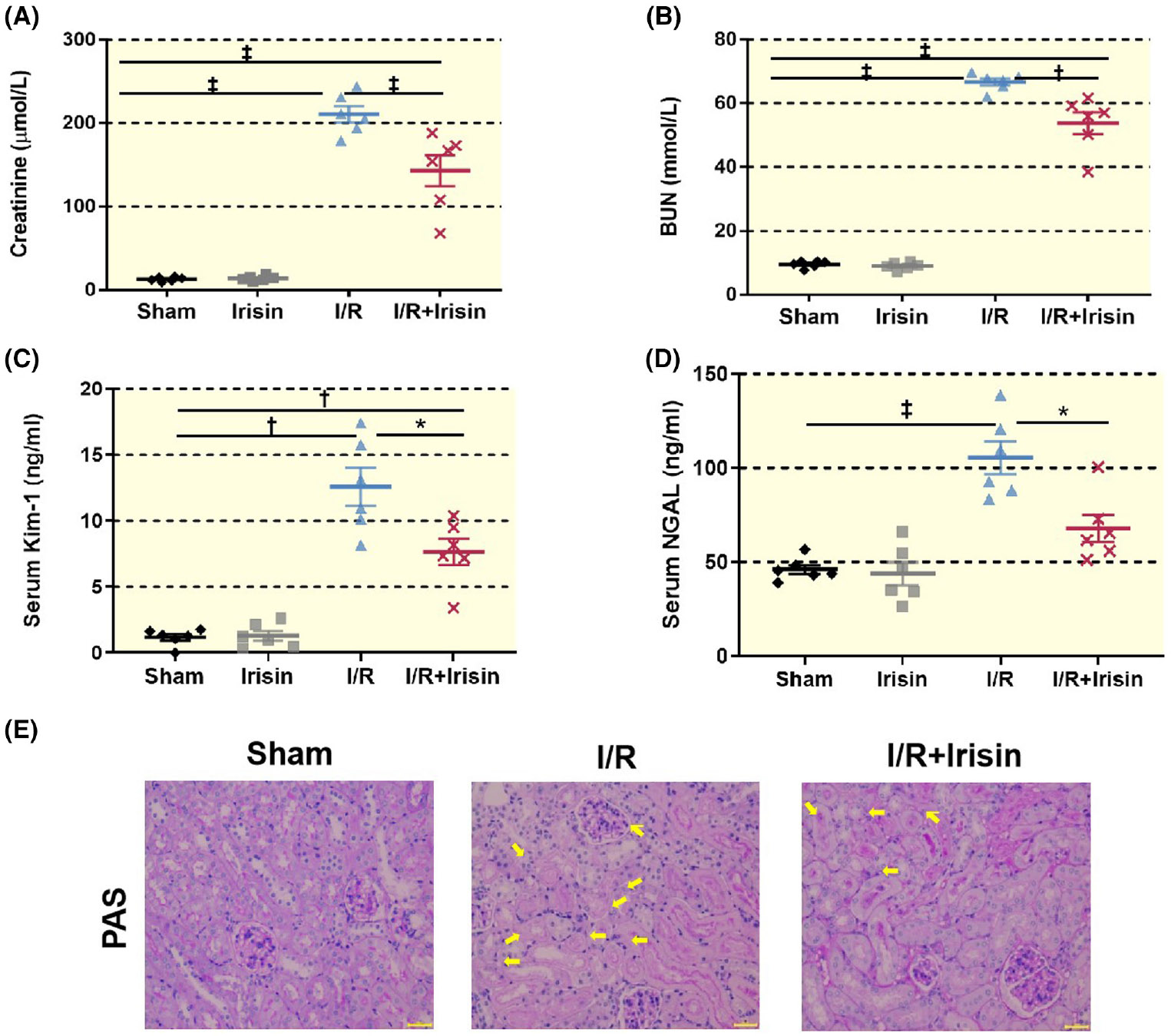
Therapeutic protective effect of exogenous irisin supplementation on renal I/R injury in mice. (A) The serum creatinine level (μmol/L) in the I/R group is significantly higher than for Sham group (210.7 ± 23.8 vs. 12.8 ± 2.6, ‡*p* < 0.001 vs. Sham) (*n* = 6), whereas creatinine in the I/R + irisin group significantly decreases to 143.0 ± 45.8 μmol/L (‡*p* < 0.001 vs. I/R) (*n* = 6). (B) The serum BUN level (mmol/L) of the I/R group is likewise higher than those of the Sham group (66.8 ± 2.7 vs. 9.6 ± 1.1, ‡*p* < 0.001 vs. Sham) (*n* = 6), whereas the BUN levels in the I/R + irisin group decreases significantly to 53.8 ± 8.4 mmol/L (†*p* < 0.01 vs. I/R) (*n* = 6). (C) The serum Kim-1 level (ng/mL) of mice of the I/R group is significantly greater than those of the Sham group (12.6 ± 3.5 vs. 1.2 ± 0.6, †*p* < 0.01 vs. Sham) (*n* = 6), whereas the Kim-1 level in the I/R + irisin group significantly decreases to 7.7 ± 2.4 ng/mL (*p < 0.05 vs. I/R) (*n* = 6). (D) The serum NGAL level of mice in the I/R group is significantly higher than that of the Sham group (105.5 ± 21.4 vs. 46.1 ± 5.9, ‡*p* < 0.001 vs. Sham) (*n* = 6). NGAL level in I/R + irisin group decreases significantly to 68.0 ± 17.6 mmol/L (*p < 0.05 vs. I/R) (*n* = 6). Statistical difference in (A–D) was calculated by *t*-test for two groups, and one-way ANOVA followed by multiple comparisons post hoc test. (E) Renal histopathology shows I/R damage and necrosis of the renal tubular epithelium. Compared to the I/R group, the I/R + irisin group exhibited less tubular injury characterized by tubular cell depletion, tubular dilation, cast formation in the tubular lumens, and loss of brush border. These data suggest irisin reduced renal tubular injury. Yellow arrows indicate injured tubules. (PAS, Bar = 50 μm, X400).

Mice in the I/R group have higher levels of serum creatinine, BUN, and kidney injury marker molecules Kim-1 and NAGL compared to the Sham group. Irisin improved renal function indicators and injury markers significantly decreased after I/R (Figure 2A-D). Accordingly, the I/R + irisin group showed less renal tubular injury as compared to the I/R group. Compared to the I/R group, the I/R + irisin mice exhibited less tubular injury characterized by tubular cell depletion, tubular dilation, cast formation in the tubular lumens, and loss of brush border (Figure 2E). These data suggest that irisin reduced renal tubular injury.

### 2.3 ∣ Mechanisms of irisin action in AKI

**FIGURE 3:**
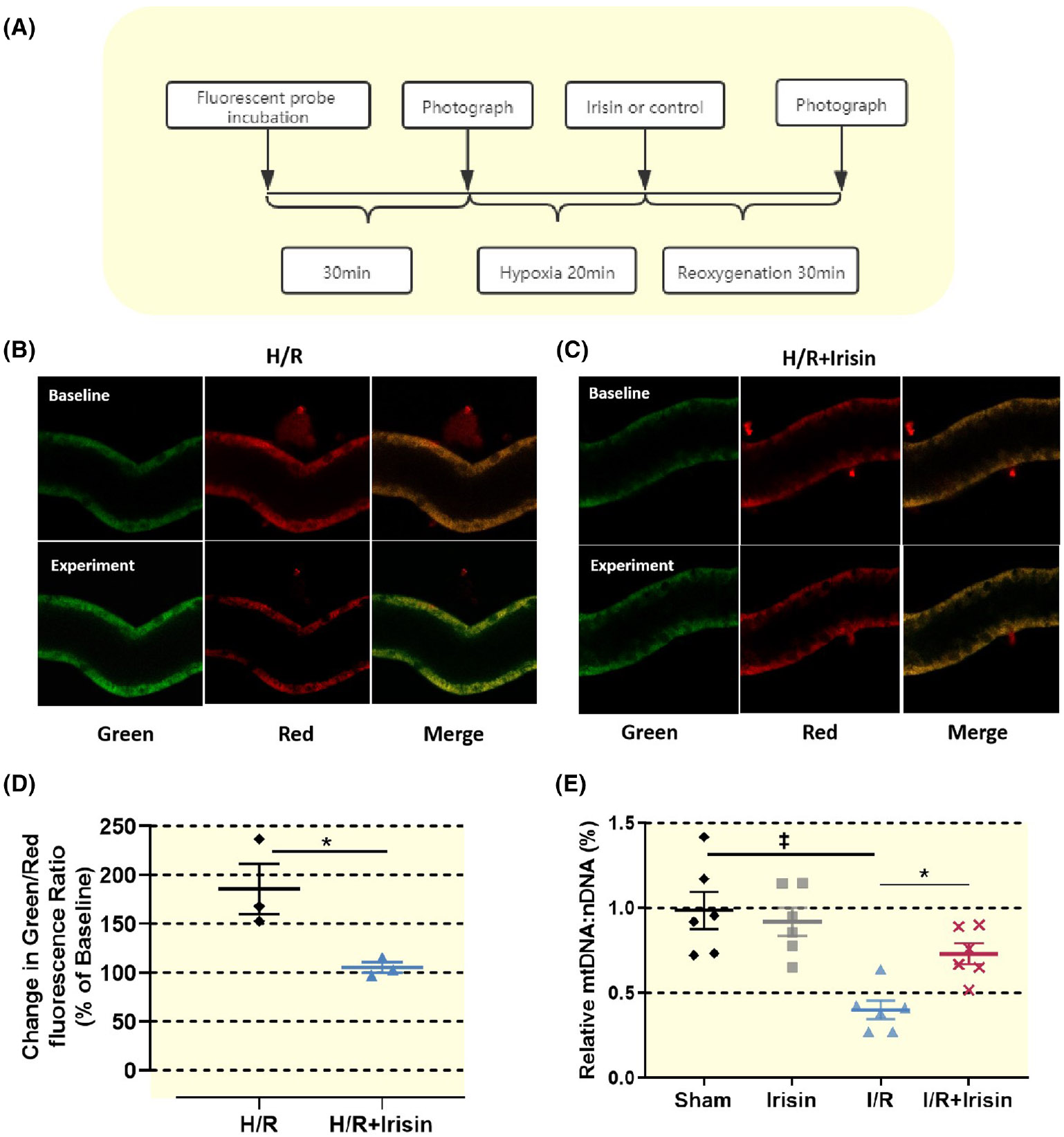
Irisin had therapeutic protective effects on renal tubular mitochondrial damage after I/R or H/R injury. (A) Flow chart of in vitro renal tubular microperfusion experiment. (B, C) When the mitochondrial membrane potential is high, JC-1 accumulates in the mitochondrial matrix to form a polymer, which can produce red fluorescence. When the membrane potential of mitochondria is low, JC-1 cannot accumulate. JC-1 is a monomer that produces green fluorescence. Under H/R, the mitochondrial membrane potential of renal tubular cells significantly decreases, while the maintenance of mitochondrial membrane potential in the irisin-treated group significantly increases. (B–D) Representative images of Green/Red fluorescence assay (B, C) and quantitative result (D) in the H/R group and the H/R + irisin group. Each experiment was repeated at least three times independently with similar results obtained. Scale bar, 50% of Baseline. (E) The mitochondrial DNA in the kidney tissue of mice in the I/R group decreases significantly compared to the Sham group (‡*p* < 0.001 vs. Sham). Irisin reduces mitochondrial DNA proportion and decreases mitochondrial damage (*p < 0.05 vs. irisin, †*p* < 0.01 vs. I/R + irisin) (*n* = 6). Statistical difference in (D–E) was calculated by *t*-test for two groups, and one-way ANOVA followed by multiple comparisons post hoc test.

Irisin alleviated mitochondrial damage: Damage to renal mitochondria was less in the I/R + irisin group compared to the I/R group. After hypoxic–reperfusion (H/R) injury in isolated active renal tubules, irisin administration into the chamber reduced mitochondrial damage (Figure 3B-E).

**FIGURE 4:**
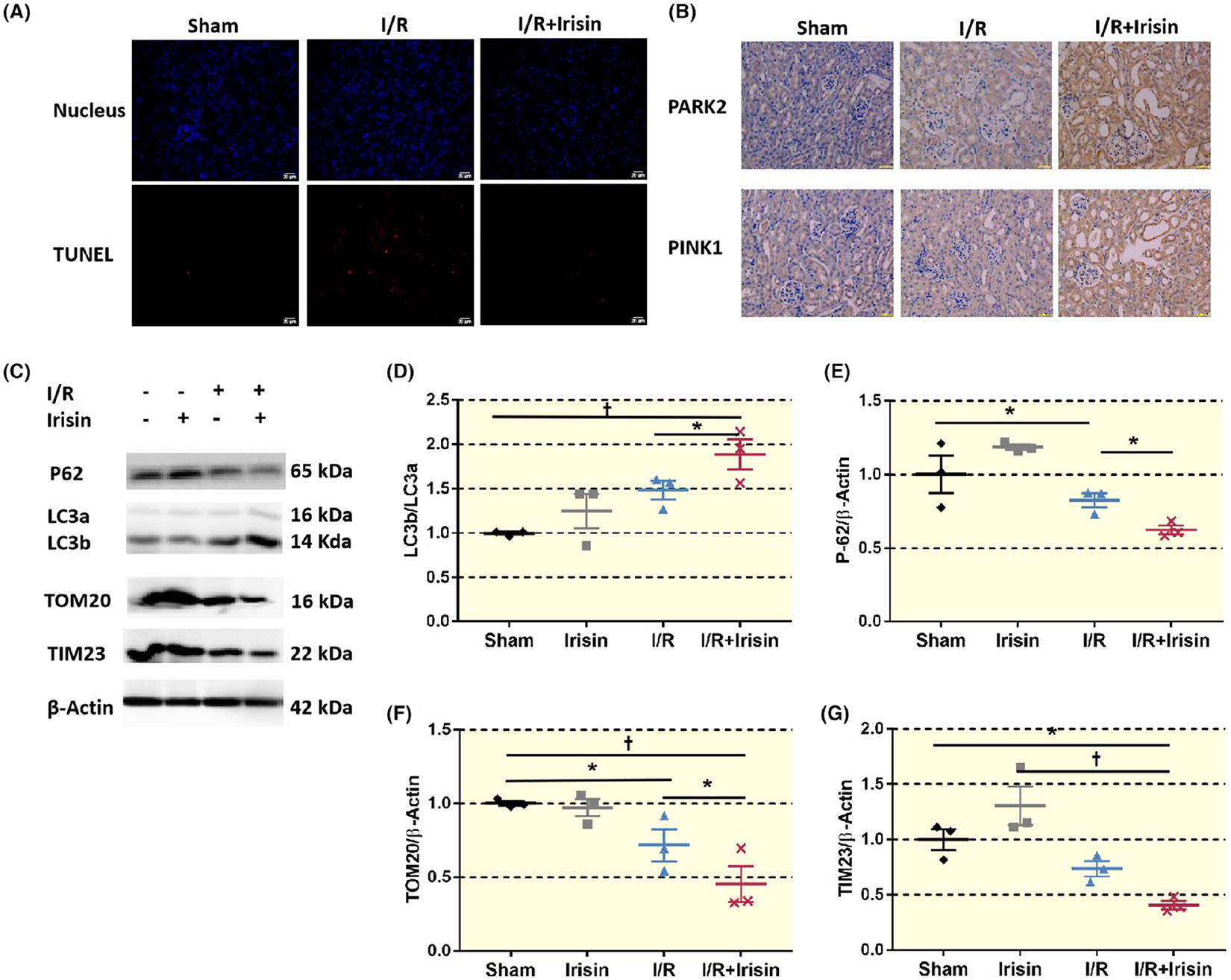
Irisin enhanced mitochondrial autophagy after renal I/R injury. (A) TUNEL staining of renal tubular cells in each group shows a significant decrease in the proportion of cell apoptosis in the I/R + irisin group compared to the I/R group. Irisin reduces renal tubular cell apoptosis (bar = 20 μm). (B) Immunohistochemistry shows increased PARK2 and PINK1 expression in the I/R group compared to Sham group. Irisin further enhances PARK2 and PINK1 expression after I/R, which is mainly enriched in renal tubular cells (×400, bar = 50 μm). C-G. LC3 is a marker protein for mitochondrial autophagy, and its increased expression indicates an enhancement of mitochondrial autophagy function. P62 is the substrate of mitochondrial autophagy, and its expression decreases with the enhancement of mitochondrial autophagy. TIM23 and TOM20 are inner mitochondrial membrane and outer membrane proteins respectively, and their contents decrease when mitochondrial autophagy increases. Western blot results show that compared to the Sham group, the I/R group shows an increase in LC3 expression, whereas the expression of P62, TOM20, and TIM23 decreases. The I/R + irisin group shows a higher expression of LC3 compared to I/R group (*p < 0.05 vs. I/R), whereas the expression of P62, TOM20, and TIM23 is lower (*p < 0.05 vs. I/R, †*p* < 0.01 vs. I/R) (*n* = 6). Statistical difference in (D–G) is calculated by *t*-test for two groups, and one-way ANOVA followed by multiple comparisons post hoc test.

Irisin enhances mitochondrial autophagy: Compared to Sham group, both I/R and irisin upregulated mitochondrial autophagy marker protein LC3 and the mitochondrial autophagy pathway-related proteins PINK1 and PARK2. Reactive substrate protein P62 and mitochondrial membrane proteins TOM20 and TIM23 were downregulated. Compared to I/R group, I/R + irisin group also upregulated LC3, PINK1 and PARK2 and downregulates P62, TOM20 and TIM23. Thus, key mitochondrial autophagy component responses seemed to indicate greater mitochondrial autophagy with less mitochondrial damage for irisin-treated mice (Figure 4).

**FIGURE 5:**
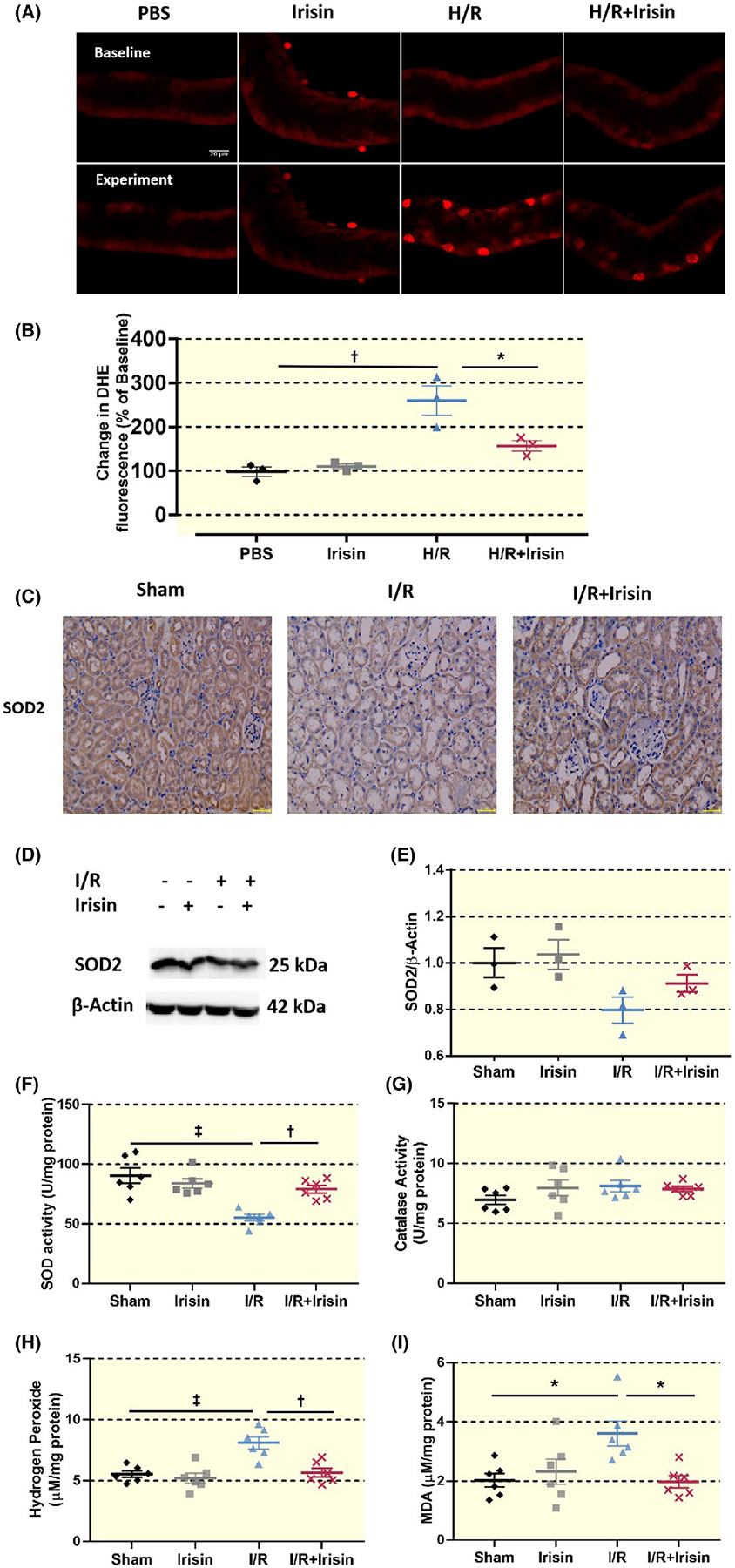
Irisin reduced ROS production after renal I/R injury. (A) DHE can freely enter the cell through the cell membrane to be oxidized by intracellular ROS, forming oxidized ethylpyridine. Oxidized ethylpyridine can be incorporated into chromosome DNA to generate red fluorescence. The content and changes of intracellular ROS are determined based on the red fluorescence production. After renal tubular H/R injury, red fluorescence is significantly enhanced. In the H/R + irisin group, the red fluorescence intensity after H/R injury is reduced dramatically compared to the H/R group, indicating that irisin limits ROS production. (A, B) Representative images of DHE fluorescence assay (A) and quantitative results (B) in four groups (*n* = 3). Scale bar, 100% of Control. (C) Immunohistochemistry shows a significant decrease in the expression of SOD2 in the I/R group and an increase in the I/R + irisin group. (D, E) The expression of SOD2 decreases during I/R, whereas the expression increases in the I/R + irisin group. (F) The SOD activity in the I/R group decreases compared to the Sham group (‡*p* < 0.001 vs. Sham), whereas the I/R + irisin group increases compared to the I/R group (†*p* < 0.01 vs. I/R) (*n* = 6). (G) The catalase activity in each group. (H) The hydrogen peroxide content in the I/R group increases as compared with Sham (‡*p* < 0.001 vs. Sham), whereas the hydrogen peroxide content in the I/R + irisin group decreases compared to the I/R group(†*p* < 0.01 vs. I/R) (*n* = 6). (I) The MDA content in the I/R group increases compared to the Sham group (*p < 0.05 vs. Sham), and the MDA content in the I/R + irisin group decreases (*p < 0.05 vs. I/R) (*n* = 6). Statistical difference in (B, E–I) is calculated by *t*-test for two groups, and one-way ANOVA followed by multiple comparisons post hoc test.

Irisin reduced renal ROS production: By renal tubular microperfusion, we tested whether irisin reduced H/R injury by limiting ROS production. Renal tubular ROS production increased by H/R. However, in the H/R + irisin group, we found less tubular ROS production when compared with the former. In the whole animal, the I/R + irisin group exhibited decreased expression of hydrogen peroxide and malondialdehyde (MDA) and increased expression of SOD to the I/R group. Thus, irisin appeared to reduce ROS production by ischemia–reperfusion both in vitro and in vivo (Figure 5).

### 2.4 ∣ Irisin reduced the activation of NLRP3 inflammasome and downstream pro-inflammatory factor release

**FIGURE 6:**
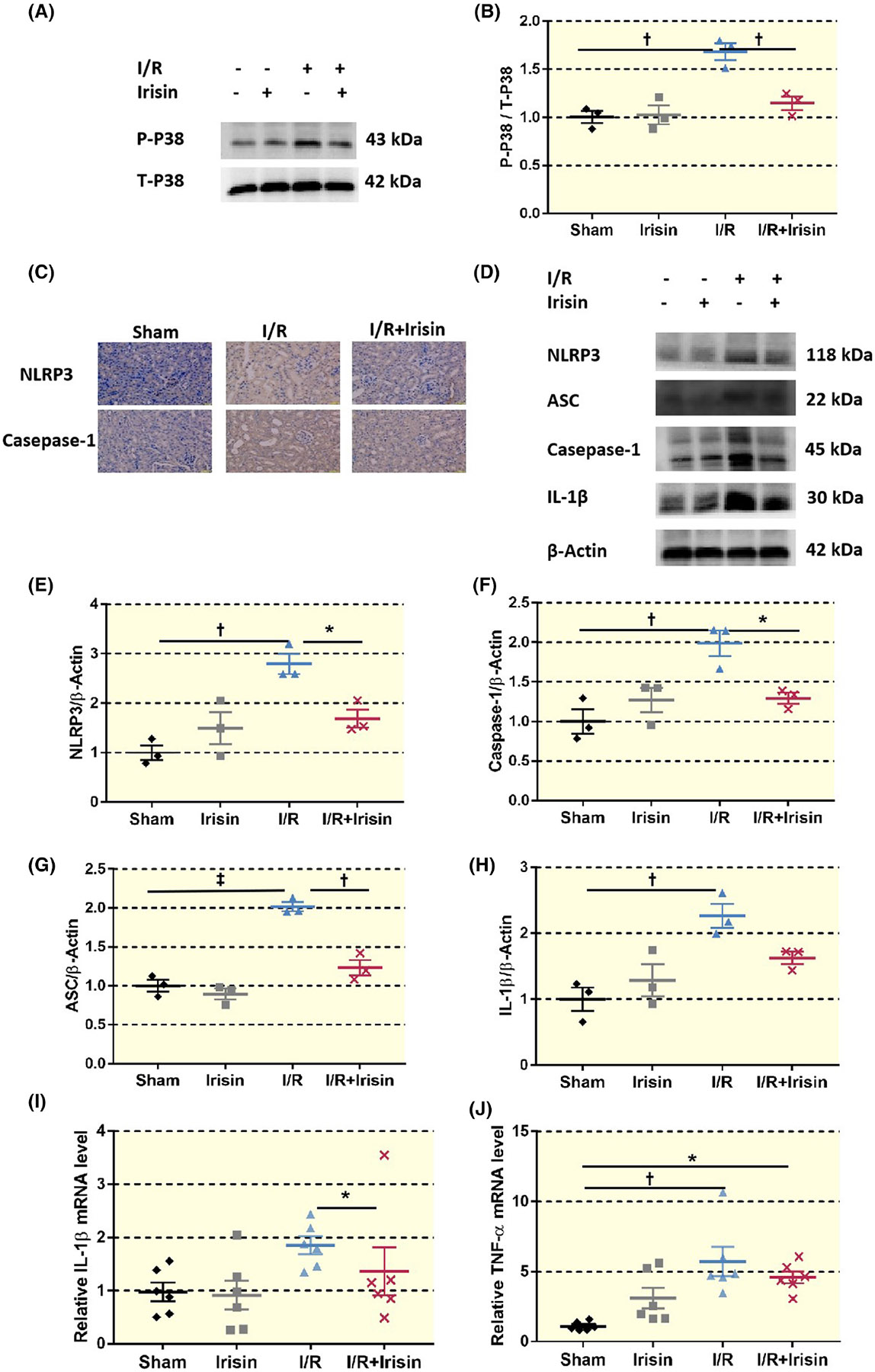
Irisin reduced NLRP3 inflammasome activation and related inflammatory factor expression after renal I/R injury. (A, B) Western blot results show an increase in renal P38 expression for the I/R group compared to the Sham group, whereas I/R + irisin shows a decrease compared to I/R (*n* = 6). (*p < 0.05 vs. I/R) Statistical difference in B is calculated by *t*-test. (C) Immunohistochemistry: Expression of NLRP3 and caspase-1 in the I/R group increases compared to sham treatment, whereas the renal content in the I/R + irisin group decreases compared to I/R (×400, bar = 50 μm). (D–H) Western blot results show that the NLRP3, ASC, caspase-1, and IL-1β levels in the kidneys of the I/R group are significantly elevated in comparison to the Sham group, whereas I/R + irisin decreases compared to the I/R group, (*n* = 6). (I, J) qRT-PCR indicates that IL-1β and TNF-α in the kidney tissue of the I/R group are higher than for Sham, whereas those of the I/R + irisin group were lower than the I/R group (*n* = 6). Statistical difference in (B, E–J) is calculated by *t*-test for two groups, and one-way ANOVA followed by multiple comparisons post hoc test.

We further investigated whether irisin could inhibit the downstream inflammatory response caused by ROS and thereby alleviate renal injury. The I/R + irisin group showed less expression of kidney inflammation-related proteins, such as P38, NLRP3, and NLRP3 inflammasome-related proteins, including ASC; and caspase-1 compared to the I/R group. Moreover, inflammatory factors IL-1β and TNF-α were reduced in the I/R + irisin group compared to the I/R group. Irisin might limit pro-inflammatory factors by attenuating NLRP3 inflammasome activation, ultimately alleviating renal I/R injury (Figure 6).

## 3 ∣ DISCUSSION

Irisin protects against I/R injury of various organs.^10,16,18,19^ This study assesses whether the kidney may be included in the group of organs potentially benefitting from irisin supplementation after an ischemic event.

**FIGURE 7:**
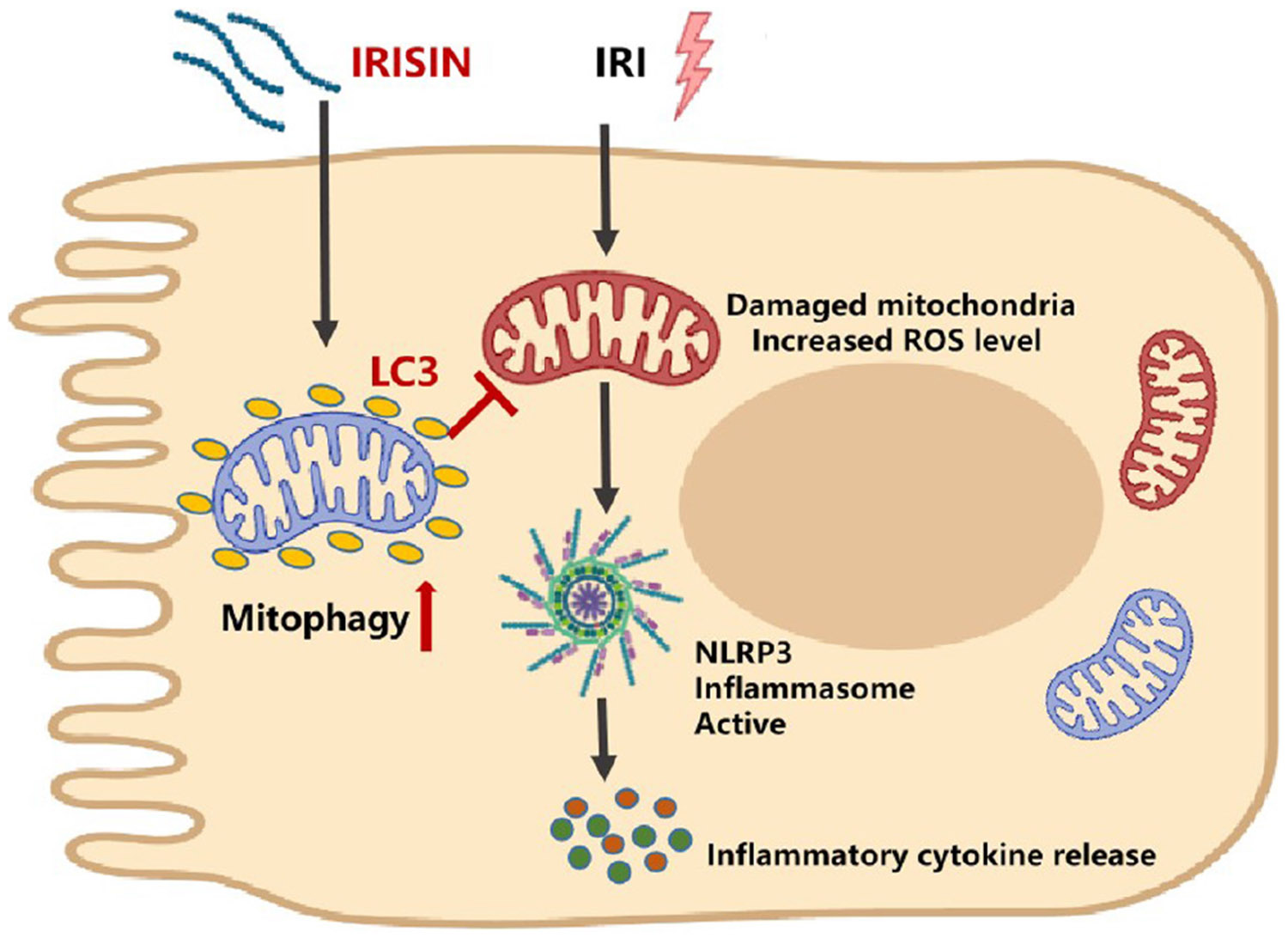
The hypothesis of the mechanism by which irisin alleviates renal I/R injury.

Patients with ischemic AKI show significantly decreased irisin concentrations in the serum, which correlate inversely with their renal function markers. When supplementing irisin after renal I/R injury in a mouse model, renal injury is decreased. Mechanistically, the presented data are compatible with the view that irisin enhances mitochondrial autophagy to clear damaged mitochondria. In consequence, ROS production decreases and NLRP3 validation body activation lessens with the effect of lowering downstream pro-inflammatory factors TNF-α and IL-1β (Figure 7).

Irisin is a multifunctional protein secreted by skeletal muscles, first discovered and named by Bostrom et al.^11^ Irisin is a matter of extensive study due to its association with chronic metabolic diseases such as obesity and diabetes.^25-27^ Circulating irisin is related to various disease states, including but not limited to, type 2 diabetes, CKD, and hypothyroidism.^28-31^ Irisin may also serve as a biomarker for acute myocardial infarction and a clinical prognostic indicator for acute pancreatitis.^16,18^ The present study shows reduced serum levels of irisin in patients with ischemic AKI. What is more, patient irisin levels correlate inversely with their renal function markers raising the question, of whether serum irisin concentration may serve as a biomarker for ischemic AKI and its severity as well.

Irisin also has anti-inflammatory, anti-apoptotic, and antioxidant effects in various settings.^32^ In an acute lung injury mouse model, irisin administration preserves the alveolar epithelium.^33^ Accordingly, in acute stroke models, irisin reduces neuronal damage and improves cognitive function by activating protein kinase B (Akt) and extracellular signal-regulated kinases 1/2 (ERK1/2) signaling pathways.^34^

Concerning the kidney, irisin inhibits the epithelial–mesenchymal transition (EMT) to lessen fibrosis.^35^ Moderate exercise in patients with chronic kidney disease significantly increases serum irisin levels and delays CKD progression.^28^ Furthermore, preventive administration of irisin before AKI in mice can have a protective effect, and its mechanism may be related to uncoupling protein 2 (UCP-2).^36^ Irisin seems affected by sex since higher plasma irisin levels are observed in men than women.^37,38^ However, the present study shows no such difference in either group (Figure 1). There are potential confounders that may influence irisin levels, such as the sample size, age, and activity intensity of patients included.^14^

Diagnosing ischemic AKI early is challenging and improved therapy is much desired. In this study, we share irisin's therapeutic effect on renal I/R injury in a mouse model. The pathophysiology of renal I/R injury appears to be characterized by a complex cascade of oxidative damage contributing to tubular injury with ultrastructural changes in mitochondria.^39,40^ Tang et al. showed that PINK1-parkin RBR E3 ubiquitin protein ligase (PRKN)-mediated mitophagy plays an important role in mitochondrial quality control, tubular cell survival, and renal function in both in vitro and in vivo models of ischemic AKI.^41^ The mechanism underlying irisin's protective effect on remote target organs is incompletely understood.^19^ The irisin receptor αV integrin was recently detected on the cell membrane, supporting irisin's potential as a circulating factor.^14^ Irisin regulates gene expression, mitosis, cell metabolism, apoptosis, and differentiation through mitogen-activated protein kinases (MAPKs), P38/ERK1/2, glucose transporter 4 (GLUT4), UCP1 mitochondria, and other pathways.^15,20,25^ However, the specific regulatory network mechanism by which irisin exerts therapeutic effects in renal I/R injury remains unclear. The data presented here indicate a possible irisin effect by enhancing mitochondrial autophagy, reducing ROS production, and inhibiting NLRP3 inflammasome activation. Mitochondria are most abundant in renal tubular cells.^42^ They play a crucial role in ischemic hypoxia necrosis of renal tubular cells, which is a key feature of ischemic AKI.^43,44^

The best-described pathway inducing mitophagy is driven by PINK1 and PRKN. The vast majority of the studies investigating the role of mitophagy in physiological or pathological conditions focus on the PINK1-PRKN pathway.^45^ Mitochondrial autophagy selectively degrades damaged or malfunctioning mitochondria through receptor protein signaling pathways such as PINK1 and directed fusion with lysosomes mediated by LC3.^46,47^ Therefore, mitochondria uphold cellular metabolism and survival. Both I/R and irisin cause autophagy activation which induced a significant increase in PINK1, PARK2, and LC3 and a decrease in P62 expression through the PINK1-PRKN pathway. It also induced a significant decrease in mitochondrial membrane marker proteins TIM23 and TOM20 (Figure 4). Enhancing mitochondrial autophagy may be an important therapeutic target for alleviating ischemic and hypoxic damage to various organs and delaying aging.^46,48^ Irisin mitigates damage by high glucose concentrations by mitochondrial autophagy through the adenosine monophosphate-activated protein kinase (AMPK)/sirtuin 1 (SIRT1)/peroxisome proliferator activated receptor-γco-activator (PGC) pathway.^49^ In hypertrophic cardiomyopathy, irisin induces LC3 expression upregulation and mitochondrial autophagy.^50^ We used renal tubular microperfusion to show that irisin alleviates mitochondrial damage and reduces ROS production in renal tubular cells after H/R injury. Irisin appears to maintain mitochondrial function by enhancing mitochondrial autophagy via upregulating the expression of related proteins PINK1 and PARK2, as well as the marker protein LC3. By reducing downstream NLRP3 inflammasome activation, irisin decreased the levels of inflammatory factors such as TNF-α and IL-1β.

A major limitation of this study is the still incompletely identified mechanisms of action. Studies using gene knockout mice or inhibitors may enhance our understanding of the interplay between ROS, mitochondrial autophagy, and NLRP3 inflammasome activation. In addition, although the histological outcomes were analyzed in a blinded fashion, the delivery of samples was not blinded. As injury severity is critically dependent on the duration of ischemia, operator experience, etc., are potential confounders.

## 4 ∣ MATERIALS AND METHODS

This study was approved by the research ethics committee at the First Affiliated Hospital, College of Medicine, Zhejiang University (No: IIT2020-789).

### 4.1 ∣ Correlation between peripheral serum irisin level and renal injury in patients with ischemic AKI

This study recruited 37 donations from January 1, 2018 to October 30, 2020 in the First Affiliated Hospital of Zhejiang University Medical College's Renal Disease Center. Informed consent was obtained from all the patients' relatives after fully explaining the purpose, nature, and risk of all procedures used. Authorized family members provided consent for organ donation. They also signed informed consent to participate in clinical trials themselves. The AKI group comprised 37 individuals who experienced ischemic AKI, but had not received continuous renal replacement therapy (CRRT) before donation. They were enrolled after cardiac death (DCD) or brain death (DBD). Inclusion criteria were age older than 18 years; diagnosis of ischemic AKI; increase in SCr ≥0.3 mg/dL (≥26.5 μmol/L) within 48 h or increase in SCr ≥1.5 times baseline, which was known or presumed to have occurred within the prior 7 days or urine volume<0.5 mL/kg/h for 6 h. Exclusion criteria were suspected diagnosis of drug-induced AKI; end-stage renal failure or renal transplantation; and suspected COVID-19 infection. The Control group comprised 20 individuals who were relatives of kidney transplant donors and had preserved peripheral blood samples before surgery. Inclusion criteria were age older than 18 years; SCr between 6 and 1.3 mg/dL; glomerular filtration rate (GFR) between 90 and 120 mL/min. Exclusion criteria were hematuria or proteinuria; history of AKI or CKD; and suspected COVID-19 infection. The study collected demographic data, clinical information, and renal function indicators. The blood and kidney biopsy (renal cortex) samples were collected from cadaveric donors during organ procurement in the Kidney Disease Center of the First Affiliated Hospital, Zhejiang University School of Medicine under the approval of the Institution Review Board (IRB) from First Affiliated Hospital, College of Medicine, Zhejiang University. Samples were immediately cooled to 4°C and centrifuged at 1000*g* for 10 min at 4°C. The serum was collected and stored at −70°C until analyzed. The concentrations of serum creatinine and BUN were measured with an autoanalyzer (Cobas 8000; Roche, Mannheim, Germany). The serum was diluted 1000 times in this experiment and the protein levels of serum irisin were assayed by an ELISA kit (Cloud-Clone Company).

### 4.2 ∣ Therapeutic effect and mechanism of irisin on renal I/R injury in mice

All animal experiments were conducted under the guidelines of the Animal Protection and Ethics Committee of the Experimental Animal Center of Zhejiang University School of Medicine and approved by the committee (No: 2020-1556).

#### 4.2.1 ∣ Animal study experimental protocol

Male C57BL/6 mice (25 ± 3 g, 8–10 weeks, Shanghai Slake Experimental Animal Company) were anesthetized with pentobarbital (50 mg/mL ip), and body temperature was controlled at 36.8–37.2°C during surgery with a temperature controlled operating table (Vestavia Scientific). Mice were randomly assigned to four mutually exclusive groups. In the ischemic kidney injury model (I/R group), mice's bilateral kidney pedicles were exposed and clamped to induce ischemia for 18 min, followed by 24 h of reperfusion. Irisin (8880-IR, R&D Systems) was preserved in a manual defrost freezer at −80°C. The dosage was 250 μg/kg body weight as reported previously.^51^ In the I/R + irisin group, following the release of clamps, irisin was reconstituted at 100 μg/mL in PBS containing 0.1% bovine serum albumin and administrated with a 29-gauge needle via penile vein injection at a single bolus of 200 μL solution (250 μg/kg body weight). In the Sham group, mice's renal pedicles were passively dissociated, but were not subjected to I/R treatment. The temperature and operation time were also controlled, and then the abdomen was closed for resuscitation; In the Sham+irisin group, mice received an injection of a single bolus of irisin (250 μg/kg) via the penile vein after sham surgery. Buprenorphine was given via subcutaneous injection at a dosage of 0.1 mg/kg for analgesia. The first dose was given at surgery and then every 8 h after surgery up to 24 h. Mice were lightly anesthetized with 1% pentobarbital, (80 mg/kg body weight) through intramuscular injection. Blood was collected into heparinized capillary tubes through the retro-orbital venous sinus. Samples were stand for 45 min in EP tube and centrifuged at 3000*g* for 10 min at 4°C and serum was collected and stored at −80°C until analyzed. Serum samples and renal tissue were taken 24 h after surgery processing as the indicators of injury severity reached the peak while the animal survival rate remained stable.

#### 4.2.2 ∣ Morphological evaluation with light microscopy

The kidneys were harvested at the end of experiments, fixed in 4% paraformaldehyde solution overnight, and then embedded in paraffin, as we have previously described.^52,53^ Kidney slices (2 μm) were cut and treated with Periodic Acid-Schiff stain (PAS). The tubular injury scoring was evaluated by two pathologists who were initially blinded to the experiment.

#### 4.2.3 ∣ Immunofluorescence

Renal tubular cells' nuclei were stained using DAPI fluorescent DNA-binding dye Kit (Abcam, UK). According to the manufacturer's instructions, renal tubular cell apoptosis was determined by terminal-deoxynucleoitidyl transferase mediated nick end labeling (TUNEL) staining using the TUNEL assay Kit (Abcam, UK). The sections were observed in the light microscope by an investigator who was initially blinded to treatment groups.

#### 4.2.4 ∣ Immunohistochemical analysis

The kidney tissue samples were immediately frozen in liquid nitrogen for protein quantitative assay and immunohistochemical analysis. Parkinson's disease 2 parkin (PARK2), PTEN-induced putative kinase 1 (PINK1), and superoxide-dismutase-2 (SOD2) in mice kidneys were detected according to the manufacturer's instructions. The 5 μm thick kidney paraffin-embedded sections cut from the tissue blocks were subjected to standard procedure for dewaxing, antigen retrieval (10 mM sodium citrate, pH 6.0), and blocking (5% BSA in PBS) before antibody staining. The tissue sections were stained with PARK2, PINK1, and SOD2 primary Abs (1:500, Abcam, USA) at 4°C overnight followed by secondary fluorescent antirabbit Abs (1:1000, ThermoFisher, USA). All images were taken using a Leica DM 4000 B scanning confocal microscope (Leica, USA) and were analyzed by an investigator who was initially blinded to the treatment groups with five randomly selected fields of each slide semi-quantified.

#### 4.2.5 ∣ Western blot analysis

Protein extracts from kidneys were separated on 7.5% SDS-PAGE gels as we have previously described.^52^ The membranes were blocked with 5% BSA for 1 h at room temperature. The primary antibodies against sequestosome 1 (P62), microtubule-associated proteins 1 light chain 3a (LC3a), microtubule-associated proteins 1 light chain 3b (LC3b), translocase of outer mitochondrial membrane 20 (TOM20), translocase of inner mitochondrial membrane 23 (TIM23), β-Actin, SOD2, phosphorylation of p38 (P-P38), P38 mitogen-activated protein kinase (T-P38) NOD-like receptor protein 3 (NLRP3), apoptosis-associated speck-like protein containing a CARD (ASC), casepase-1 and interleukin-1β (IL-1β) were diluted according to instruction (Abcam, UK). And then incubated membranes overnight (at 4°C) with antibodies. Subsequently, the membranes were washed (3 × 10 min), then incubated with a horseradish peroxidase-conjugated secondary antibody (goat anti-mouse IgG; 1:300000; Bio-Rad, Hercules, CA) for 1 h at room temperature. They were then washed again (3 × 10 min), and the immunoreactive bands were detected by the ChemiDoc System (Bio-Rad, Hercules, CA) and quantified by ImageLab software (Bio-Rad, Hercules, CA). Normalization was performed by stripping the membranes with restore western blot stripping buffer (Thermo Fisher Scientific, Waltham, MA) for 15 min at room temperature and incubating them with β-actin antibody (mouse monoclonal IgG; 1:5000; Sigma, St. Louis, MO).

#### 4.2.6 ∣ Measurement of mrna expression by qPCR

We measured the mRNA levels of inflammatory-related genes: tumor necrosis factor-α (TNF-α) and IL-1β 24 h after surgery. Total RNAs were extracted from the left kidney and digested with RNase-free DNase (Promega, Fitchburg, WI). Reverse transcription was performed to synthesize cDNA templates as we described previously.^54^ QPCR analysis was performed using iTaq Universal SYBR Green Supermix (Bio-Rad, Hercules, CA) and CFX96 Real-Time Detection System (Bio-Rad, Hercules, CA) according to the manufacturer's protocol. The reaction conditions were 95°C for 1 min, followed by 40 cycles of 95°C for 15 s and 60°C for 30 s. The comparative Ct method (2^−ΔΔCt^) was used to analyze data and calculate the relative mRNA expression levels. The primer sequences and accession numbers are listed in the supplementary material.

#### 4.2.7 ∣ Enzyme-linked immunosorbent assay of cytokines analysis

Serum Kim-1 and NGAL levels were measured using enzyme-linked immunosorbent assay (ELISA) kits (Bio-Rad, Hercules, CA) as we previously described.^55^ The detection range is 0.16–10 ng/mL and 39.06–2500 pg/mL respectively. Serum was diluted 2 times and 100 times respectively in these experiments. Then serum irisin was measured using ELISA kits (#SEN576Hu, CLOUD-CLONE Inc., Wuhan, China) according to the manufacturer's instructions. The serum was diluted 1000 times with 1% PBS. Briefly, prepare standard substances, reagents, and samples before the experiment; add 100 μL of the sample, and incubate at 37°C for 1 h; discard and add 100 μL of detection solution A, incubate at 37°C for 1 h; wash the plate 3 times with PBS; add 100 μL of detection solution B, incubate at 37°C for 30 min; wash the plate 5 times; add 90 μL of TMB substrate, incubate at 37°C for 10–20 min; add 50 μL of stop solution, immediately read at 450 nm. Samples used for ELISA assays were coded, and the laboratory investigator was initially blinded to the treatment groups.

#### 4.2.8 ∣ Tubular microperfusion

Isolation and microperfusion of proximal tubules: Briefly, mice were anesthetized with isoflurane, and kidneys were removed and sliced along the corticomedullary axis. Slices were placed in ice-cold DMEM and dissected under a stereomicroscope. A single proximal tubule attached glomerulus was isolated and then the glomerulus was cut away. Next, the proximal tubule was transferred to a temperature-controlled chamber on the stage of confocal microscopy and perfused using a micromanipulator system with concentric holding and perfusion pipettes, as we previously described.^56-58^

The measurements of the mitochondrial damage and reactive oxygen species (ROS) in proximal tubules: the isolated proximal tubule was incubated with JC-1(5 μM; Thermo Fisher Scientific, MA, USA) or dihydroethidium (DHE) (5 μM; Apexbio, TX, USA) probes in DMEM for 30 min and then washed with DMEM. During this period, the bath solution and tubular perfusate were maintained with an air-bubbled solution for 30 min and then switched to the solution bubbled with nitrogen-inducing hypoxia for 20 min, followed by administration of irisin (1 μg/mL) and reoxygenation with the air bubbled solution for 30 min. Images were captured before tubular hypoxia and right after 30 min of reoxygenation using confocal microscopy (Olympus FV500). The fluorescent intensity was quantified using Image J software.

### 4.3 ∣ Statistical analysis

Statistical analysis was performed using Prism Graph 9.0. Qualitative variables were analyzed with Fisher's exact test and Pearson's chi-squared test. Quantitative variables were analyzed with the Student's *t*-test when the data were normally distributed, whereas the Mann–Whitney test or Kruskal–Wallis test were used for non-normally distributed data. *p* < 0.05 indicates statistical significance.

## 5 ∣ CONCLUSION

This study provides a basis for testing irisin as a therapeutic drug for ischemic AKI. Irisin may serve as a candidate biomarker for ischemic AKI and its progression. Irisin's protective effect could involve enhancing mitochondrial autophagy by upregulating mitochondrial autophagy marker protein LC3 to reduce downstream NLRP3 inflammasome activation.

## Supplementary Material

Supplemental Data

## Data Availability

The data that support the findings of this study are available on request from the corresponding author. The data are not publicly available due to privacy or ethical restrictions.
