# Supplementary material for: Irisin preserves mitochondrial integrity and function in tubular epithelial cells after ischemia–reperfusion-induced acute kidney injury
Source: Acta Physiol (Oxf). Author manuscript; Available in PMC 2025 Mar 11. (PMC11894518; doi:10.1111/apha.14211)
Supplement: Supplemental Data [file NIHMS2058539-supplement-Supplemental_Data.docx]

**Supplementary Material**

**Irisin Preserves Mitochondrial Integrity and Function in Tubular Epithelial Cells after Ischemia-Reperfusion Induced Acute Kidney Injury**

Yu Cui^1^, Lu Yu^1^, Wenqi Cong^1^, Shan Jiang^2^, Xingyu Qiu^2^, Chunchun Wei^1^, Gui Zheng^1^, Jianhua Mao^3^, Ruisheng Liu^4^, Andreas Patzak^5^, Pontus B. Persson^5^, Jianghua Chen^1*^, Liang Zhao^3*^, En Yin Lai^1,2,5*^

1. Kidney Disease Center of the First Affiliated Hospital, Zhejiang University School of Medicine, Key Laboratory of Kidney Disease Prevention and Control Technology, Zhejiang Province, Zhejiang Clinical Research Center of Kidney and Urinary System Disease, Hangzhou, China
2. Department of Physiology, School of Basic Medical Sciences, Zhejiang University School of Medicine, Hangzhou, China
3. Department of Nephrology, Children’s Hospital, Zhejiang University School of Medicine, National Clinical Research Center for Child Health, Provincial Key Laboratory of Neonatal Diseases, Hangzhou, China
4. Department of Molecular Pharmacology & Physiology; Hypertension and Kidney Research Center, Morsani College of Medicine, University of South Florida, Tampa 33612, USA
5. Institute of Translational Physiology, Charité–Universitätsmedizin Berlin, corporate member of Freie Universität Berlin and Humboldt-Universität zu Berlin, Berlin 10117, Germany

**Supplemental Tables**

**Table S1. Demographic characteristic of Healthy population and AKI patients**

| Subject Characteristic | Control | AKI Group |
| --- | --- | --- |
| Number | 20 | 37 |
| Age, y | 52.2±12.52 | 44.37±12.43 |
| Sex, male,（N，%） | 7（35%）* | 29（78.4%） |
| Height (m) | 1.65±0.11 | 1.63±0.10 |
| Weight (kg) | 58.5±7.8 | 59.3±8.3 |
| Body mass index (kg/m^2^) | 22.16±7.35 | 20.88±9.45 |

**Table S1：**Data are mean ± SEM. * p<0.05 vs AKI. Statistical difference was calculated by paired t-test.

Table S2. Major Resources

**Primary Antibodies**

| **Target antigen** | **Host species** | **Vendor or Source** | **Catalog#** | **Working concentration** | **Persistent ID/ URL** |
| --- | --- | --- | --- | --- | --- |
| P62 | Rabbit | Abcam | ab109012 | 1:10000(WB) | https://www.abcam.cn/products/primary-antibodies/sqstm1--p62-antibody-epr4844-autophagosome-marker-ab109012.html |
| LC3a/b | Rabbit | Abcam | ab62721 | 1:2000(WB) | https://www.abcam.cn/products/primary-antibodies/lc3ab-antibody-ab62721.html |
| TOM20 | Rabbit | Abcam | ab186735 | 1:1000(WB) | https://www.abcam.com/products/primary-antibodies/tomm20-antibody-epr15581-54-mitochondrial-marker-ab186735.html |
| T2M23 | Rabbit | Abcam | ab230253 | 1:1000(WB) | https://www.abcam.com/products/primary-antibodies/timm23tim23-antibody-ab230253.html |
| β-Actin | Mouse | Abcam | ab8226 | 1 µg/ml(WB) | https://www.abcam.com/products/primary-antibodies/beta-actin-antibody-mabcam-8226-loading-control-ab8226.html?productWallTab=Abreviews |
| SOD2 | Rabbit | Abcam | ab68155 | 1:1000(WB) | https://www.abcam.com/products/primary-antibodies/sod2mnsod-antibody-epr2560y-ab68155.html |
| P38 P-AMPK | Rabbit | Abcam | Ab17099 | 1:1000(WB) | https://www.abcam.com/products/primary-antibodies/p38-alphamapk14-antibody-e229-ab170099.html |
| NLRP3 | Rabbit | Abcam | Ab263899 | 1:1000(WB) | https://www.abcam.com/products/primary-antibodies/nlrp3-antibody-epr23094-1-ab263899.html |
| ASC | Rabbit | Abcam | Ab283684 | 1:1000(WB) | https://www.abcam.com/products/primary-antibodies/tms1asc-antibody-epr23978-28-ab283684.html |
| Casepase-1 | Rabbit | Abcam | Ab207802 | 1:1000(WB) | https://www.abcam.com/products/primary-antibodies/caspase-1-antibody-epr19672-ab207802.html |
| IL-1β | Rabbit | Abcam | Ab254360 | 1:1000(WB) | https://www.abcam.com/products/primary-antibodies/il-1-beta-antibody-epr23851-127-ab254360.html |
| Kim-1 | Mouse | Elabscience | E-EL-M3039 | NA（ELISA） | https://www.elabscience.com/p-mouse_kim_1_kidney_injury_molecule_1_elisa_kit-356285.html |
| NAGL | Mouse | Elabscience | E-EL-M0828 | NA（ELISA） | https://www.elabscience.com/p-mouse_ngal_neutrophil_gelatinase_associated_lipocalin_elisa_kit-20785.html |
| BrdU | Mouse | Abcam | ab6326 | 1：250（IF） | https://www.abcam.com/products/primary-antibodies/brdu-antibody-bu175-icr1-proliferation-marker-ab6326.html |
| DAPI | / | Abcam | Ab285390 | NA |  |

**Secondary Antibody**

| **Target antigen** | **Host species** | **Vendor or Source** | **Catalog#** | **Working concentration** | **Persistent ID/URL** |
| --- | --- | --- | --- | --- | --- |
| Mouse IgG  (H+L) | Goat | Bio-Rad, Hercules, CA | #1706516 | 1:300,000 | https://www.bio-rad.com/zh-cn/sku/1706516-goat-anti-mouse-igg-h-l-hrp-conjugate?ID=1706516 |
| Goat  IgG | Mouse | Sigma, St. Louis, MO | B3148 | 1:5000 | https://www.sigmaaldrich.com/US/en/product/sigma/b3148 |

**NGAL kit Inter and intraassay variation**

|  | **Intra-assay Precision** | | | **Inter-assay Precision** | | |
| --- | --- | --- | --- | --- | --- | --- |
| Sample | 1 | 2 | 3 | 1 | 2 | 3 |
| n | 20 | 20 | 20 | 20 | 20 | 20 |
| Mean (pg/mL) | 119.40 | 390.07 | 1093.66 | 121.64 | 352.20 | 1131.18 |
| Standard deviation | 6.93 | 17.28 | 43.64 | 7.99 | 18.81 | 60.41 |
| CV (%) | 5.80 | 4.43 | 3.99 | 6.57 | 5.34 | 5.34 |

**Kim-1 kit Inter and intraassay variation**

|  | **Intra-assay Precision** | | | **Inter-assay Precision** | | |
| --- | --- | --- | --- | --- | --- | --- |
| Sample | 1 | 2 | 3 | 1 | 2 | 3 |
| n | 20 | 20 | 20 | 20 | 20 | 20 |
| Mean (ng/mL) | 0.42 | 1.04 | 4.86 | 0.47 | 1.16 | 4.38 |
| Standard deviation | 0.02 | 0.05 | 0.22 | 0.03 | 0.06 | 0.22 |
| CV (%) | 4.76 | 4.81 | 4.53 | 6.38 | 5.17 | 5.02 |

**Oligonucleotides**

| **Primer Name** | **Sequence** | **Use** |
| --- | --- | --- |
| TNF-α F | GATCGGTCCCCAAAGGGATG | qRT-PCR |
| TNF-α R | GGTGGTTTGTGAGTGTGAGGG | qRT-PCR |
| IL-1β F | TGCCACCTTTTGACAGTGAT | qRT-PCR |
| IL-1β R | CTGCCTGAAGCTCTTGTTGA | qRT-PCR |
| GAPDH F | AAATGGTGAAGGTCGGTGTGAAC | qRT-PCR |
| GAPDH R | CAACAATCTCCACTTTGCCACTG | qRT-PCR |

**Supplemental Figures**

**Figure S1.**

**
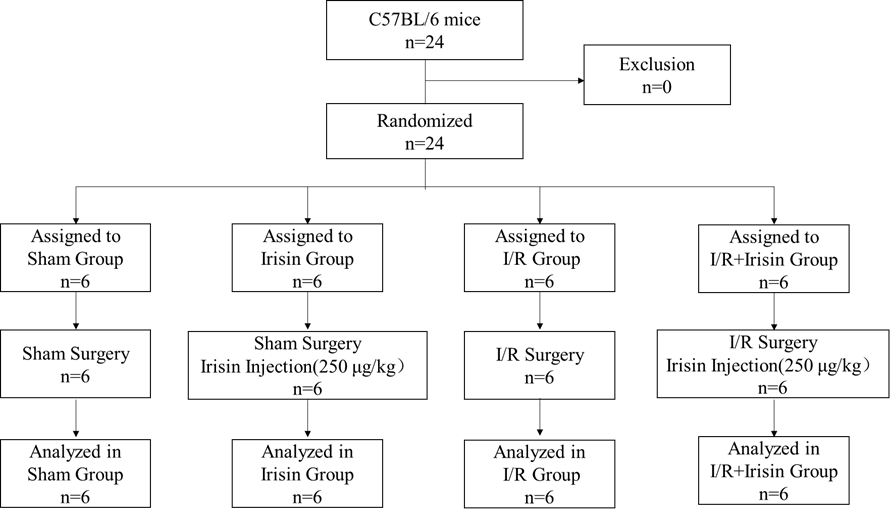
**

**Figure S1.** Consort-like diagram of animal experiments.

**Figure S2.**


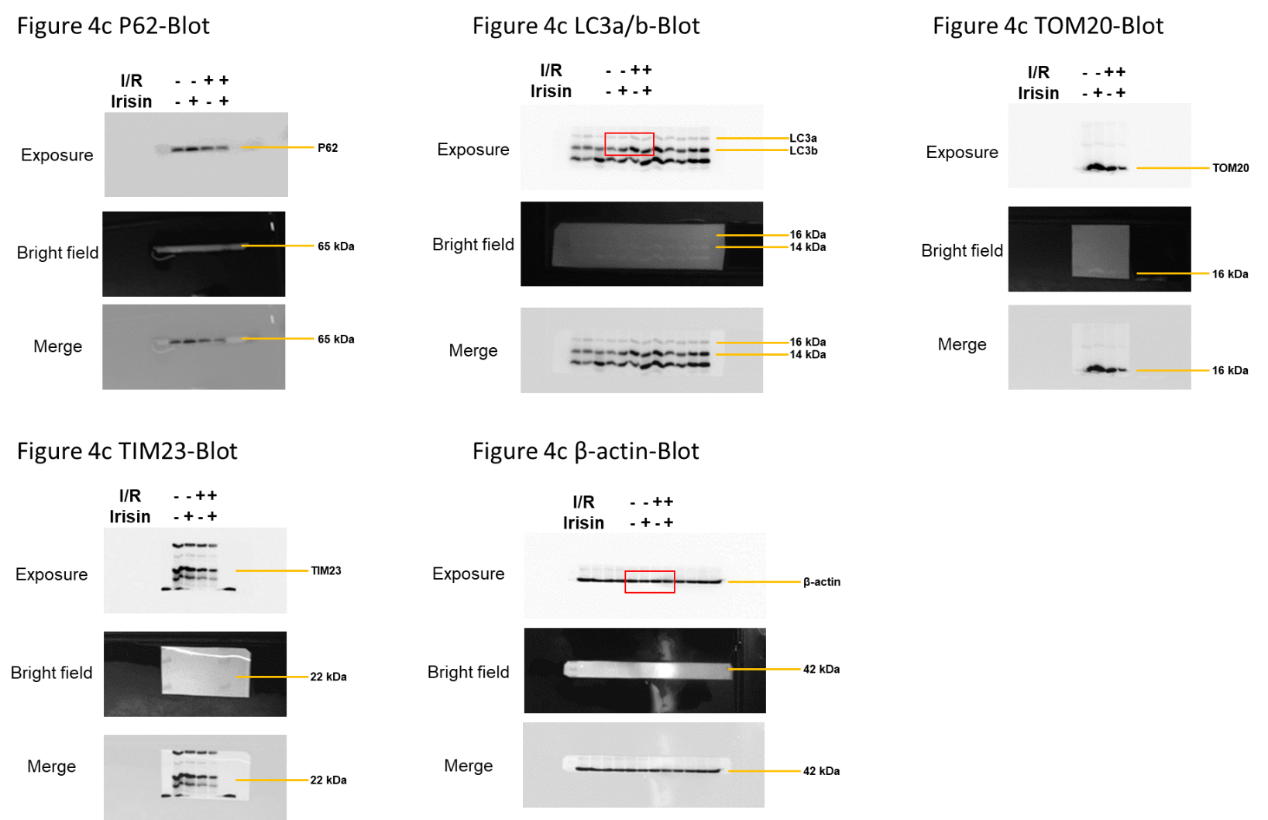


**Figure S2 Entire blot lanes including molecular markers for all cropped western blot bands of figure 4 shown in the main body of the manuscript.**

P62 is a multidomain protein with roles in the regulation of numerous signaling pathways through distinct binding partners. I/R, ischemia and reperfusion. LC3a, microtubule-associated protein 1 light chain 3a. LC3b, microtubule-associated protein 1 light chain 3a. TOM20,translocase of outer mitochondrial membrane 20. TIM23, translocase of inner mitochondrial membrane 23.

**Figure S3.**


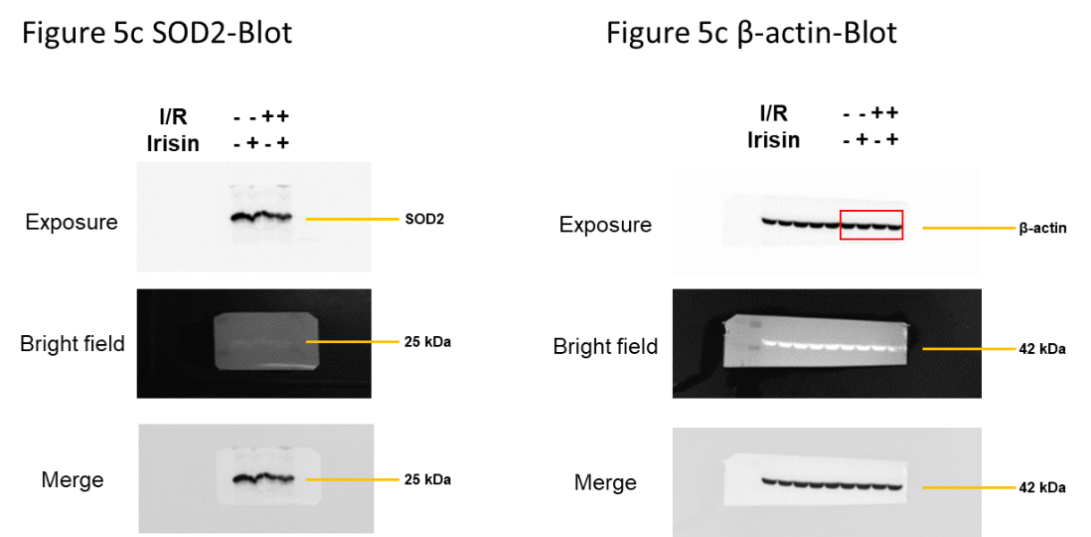


**Figure S3 Entire blot lanes including molecular markers for all cropped western blot bands of figure 5 shown in the main body of the manuscript.**

SOD2, superoxide dismutase 2.

**Figure S4.**


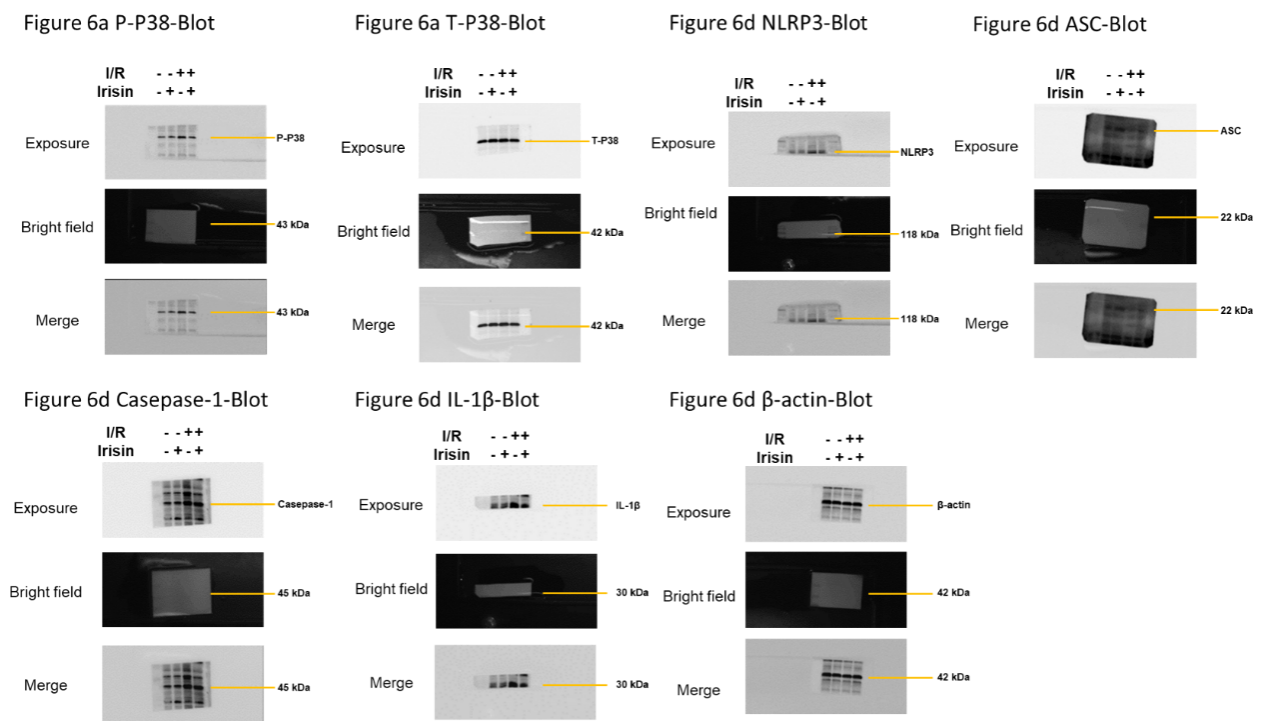


**Figure S4 Entire blot lanes including molecular markers for all cropped western blot bands of figure 6 shown in the main body of the manuscript.**

P-P38, phosphorylation of p38. T-P38, P38 Mitogen-Activated Protein Kinase. NLRP3, NOD-like receptor protein 3. ASC, Apoptosis-associated speck-like protein containing a CARD. IL-1β, interleukin-1.
